# Supplementary material for: Interactive effects of drought and deforestation on multitrophic communities and aquatic ecosystem functions in the Neotropics—a test using tank bromeliads
Source: PeerJ. 2024 May 8;12:e17346. doi: 10.7717/peerj.17346 (PMC11088369; doi:10.7717/peerj.17346)
Supplement: Supplemental Information 1 — D+Dr = deforestation and drought sequentially, D_Dr = deforestation and drought simultaneously; p-value in bold denote significant effects (p < 0.05). Estimate = contrast of means, SE = Standard Error, DF = Degree of Freedom, Lower and Upper CL = Confidence Limits for the mean [file peerj-12-17346-s001.docx]

| Univariate response | Comparison | Estimate | SE | Df | tvalue | P value |
| --- | --- | --- | --- | --- | --- | --- |
| Log(Water volume +1) (mL) | Forest control - Deforestation | -2.90 | 3.29 | 63 | -0.881 | 1.0000 |
|  | Forest control – Forest Drought | 22.70 | 3.29 | 63 | 6.900 | **6.274*10^-8^** |
|  | Forest control – D+Dr | 35.25 | 3.29 | 63 | 10.714 | **1.662*10^-14^** |
|  | Forest control – D_Dr | 37.70 | 3.29 | 63 | 11.459 | **9.917*10^-16^** |
|  | Forest control – Open Control | -15.00 | 3.29 | 63 | -4.559 | **5.084*10^-4^** |
|  | Forest control – Open drought | 18.15 | 3.29 | 63 | 5.517 | **1.451*10^-5^** |
|  | Deforestation – Forest drought | 25.60 | 3.29 | 63 | 7.781 | **1.809*10^-9^** |
|  | Deforestation – D+Dr | 38.15 | 3.29 | 63 | 11.596 | **5.951*10^-16^** |
|  | Deforestation – D_Dr | 40.60 | 3.29 | 63 | 12.341 | **3.840*10^-17^** |
|  | Deforestation – Open control | -12.10 | 3.29 | 63 | -3.678 | **1.026*10^-2^** |
|  | Deforestation – Open drought | 21.05 | 3.29 | 63 | 6.398 | **4.638*10^-7^** |
|  | Forest drought – D+Dr | 12.55 | 3.29 | 63 | 3.815 | **6.576*10^-3^** |
|  | Forest drought – D_Dr | 15.00 | 3.29 | 63 | 4.559 | **5.084*10^-4^** |
|  | Forest drought – Open control | -37.70 | 3.29 | 63 | -11.459 | **9.917*10^-16^** |
|  | Forest drought – Open drought | -4.55 | 3.29 | 63 | -1.383 | 1.0000 |
|  | D+Dr – D_Dr | 2.45 | 3.29 | 63 | 0.745 | 1.0000 |
|  | D+Dr – Open control | -50.25 | 3.29 | 63 | -15.274 | **1.561*10^-21^** |
|  | D+Dr – Open drought | -17.10 | 3.29 | 63 | -5.198 | **4.875*10^-5^** |
|  | D_Dr – Open control | -52.70 | 3.29 | 63 | -16.018 | **1.424*10^-22^** |
|  | D_Dr – Open drought | -19.55 | 3.29 | 63 | -5.942 | **2.794*10^-6^** |
|  | Open control – Open drought | 33.15 | 3.29 | 63 | 10.076 | **1.956*10^-13^** |
| Log(Light input (%)) | Forest control – Deforestation | -37.50 | 5.75 | 63 | -6.521 | **2.852*10^-7^** |
|  | Forest control – Forest Drought | 0.10 | 5.75 | 63 | 0.017 | 1.0000 |
|  | Forest control – D+Dr | -33.25 | 5.75 | 63 | -5.782 | **5.223*10^-6^** |
|  | Forest control – D_Dr | -38.85 | 5.75 | 63 | -6.755 | **1.118*10^-7^** |
|  | Forest control – Open Control | -28.20 | 5.75 | 63 | -4.904 | **1.457*10^-4^** |
|  | Forest control – Open drought | -36.60 | 5.75 | 63 | -6.364 | **5.309*10^-7^** |
|  | Deforestation – Forest drought | 37.60 | 5.75 | 63 | 6.538 | **2.661*10^-7^** |
|  | Deforestation – D+Dr | 4.25 | 5.75 | 63 | 0.739 | 1.0000 |
|  | Deforestation – D_Dr | -1.35 | 5.75 | 63 | -0.235 | 1.0000 |
|  | Deforestation – Open control | 9.30 | 5.75 | 63 | 1.617 | 1.0000 |
|  | Deforestation – Open drought | 0.90 | 5.75 | 63 | 0.156 | 1.0000 |
|  | Forest drought – D+Dr | -33.35 | 5.75 | 63 | -5.799 | **4.882*10^-6^** |
|  | Forest drought – D_Dr | -38.95 | 5.75 | 63 | -6.773 | **1.043*10^-7^** |
|  | Forest drought – Open control | -28.30 | 5.75 | 63 | -4.921 | **1.367*10^-4^** |
|  | Forest drought – Open drought | -36.70 | 5.75 | 63 | -6.382 | **4.955*10^-7^** |
|  | D+Dr – D_Dr | -5.60 | 5.75 | 63 | -0.974 | 1.0000 |
|  | D+Dr – Open control | 5.05 | 5.75 | 63 | 0.878 | 1.0000 |
|  | D+Dr – Open drought | -3.35 | 5.75 | 63 | -0.583 | 1.0000 |
|  | D_Dr – Open control | 10.65 | 5.75 | 63 | 1.852 | 1.0000 |
|  | D_Dr – Open drought | 2.25 | 5.75 | 63 | 0.391 | 1.0000 |
|  | Open control – Open drought | -8.40 | 5.75 | 63 | -1.461 | 1.0000 |
