# Supplementary material for: Interactive effects of drought and deforestation on multitrophic communities and aquatic ecosystem functions in the Neotropics—a test using tank bromeliads
Source: PeerJ. 2024 May 8;12:e17346. doi: 10.7717/peerj.17346 (PMC11088369; doi:10.7717/peerj.17346)
Supplement: Supplemental Information 2 — Feeding group categorization according to Merritt & Cummins (1978). Stars indicate species that occurred in less than 2 bromeliads, and that were removed from the analysis. [file peerj-12-17346-s002.docx]

| Class | Order | Family | Species | Feeding group |
| --- | --- | --- | --- | --- |
| Annelida | Haplotaxida | Naididae | *Aulophorus superterrenus* | Collector |
|  |  |  | *Pristina* sp.* | Collector |
|  |  | Enchytraeidae | *Enchytraeidae* sp. | Collector |
| Copepoda |  |  | Unknown* | Filter feeder |
| Insecta | Coleoptera | Hydrophilidae | Sphaeridinae* | Predator |
|  |  | Scirtidae | *Scirtes* sp. | Scraper |
|  | Diptera | Cecidomyiidae | Cecidomyiidae sp1.* | Leaf piercer |
|  |  | Ceratopogonidae | *Bezzia* sp2.* | Predator |
|  |  | Chironomidae | Chironomini | Collector |
|  |  |  | Tanypodinae sp. | Predator |
|  |  |  | *Tanytarsus* sp. | Collector |
|  |  | Corethrellidae | *Corethrella* sp. | Predator |
|  |  | Culicidae | *Microculex stonei* | Filter feeder |
|  |  |  | *Wyeomyia aphobema* | Filter feeder |
|  |  | Dolichopodidae | Unknown* | Predator |
|  |  | Psychodidae | *Telmatoscopus* sp.* | Shredder |
|  |  | Tabanidae | Unknown* | Predator |
|  |  | Tipulidae | *Trentepohlia* sp1. | Shredder |
|  |  |  | *Trentepohlia* sp2.* | Shredder |
|  | Odonata | Erythrodiplax | *Unknown* | Predator |
|  |  | Coenagrionidae | *Leptagrion sp1.* | Predator |
|  |  |  | *Leptagrion sp2.* | Predator |
| Ostracoda | Podocopida | Limnocytheridae | *Elpidium bromeliarum* | Collector |

**Reference**

Merritt, R. W., & Cummins, K. W. (Eds.). (1978). *An introduction to the aquatic insects of North America*. Kendall/Hunt Pub. Co.
