# Supplementary material for: Interactive effects of drought and deforestation on multitrophic communities and aquatic ecosystem functions in the Neotropics—a test using tank bromeliads
Source: PeerJ. 2024 May 8;12:e17346. doi: 10.7717/peerj.17346 (PMC11088369; doi:10.7717/peerj.17346)
Supplement: Supplemental Information 3 — Bolded pvalue are significant. [file peerj-12-17346-s003.docx]

| Univariate responses | Mesh size | Comparisons | W | P value |
| --- | --- | --- | --- | --- |
| Attached bacteria | Coarse | Forest control – Open control | 87 | **0.0039** |
|  | Fine | Forest control – Open control | 52 | **0.016** |
| Attached fungi | Coarse | Forest control – Open control | 53 | 0.8534 |
|  | Fine | Forest control – Open control | 50 | **0.0312** |
| Attached bacteria | Coarse | Open control – Open drought | 66 | 0.2475 |
|  | Fine | Open control – Open drought | 64 | 0.315 |
| Attached fungi | Coarse | Open control – Open drought | 68 | 0.1903 |
|  | Fine | Open control – Open drought | 33 | 0.2176 |
