# Supplementary figures and images for: Interactive effects of drought and deforestation on multitrophic communities and aquatic ecosystem functions in the Neotropics—a test using tank bromeliads
Source: PeerJ. 2024 May 8;12:e17346. doi: 10.7717/peerj.17346 (PMC11088369; doi:10.7717/peerj.17346)

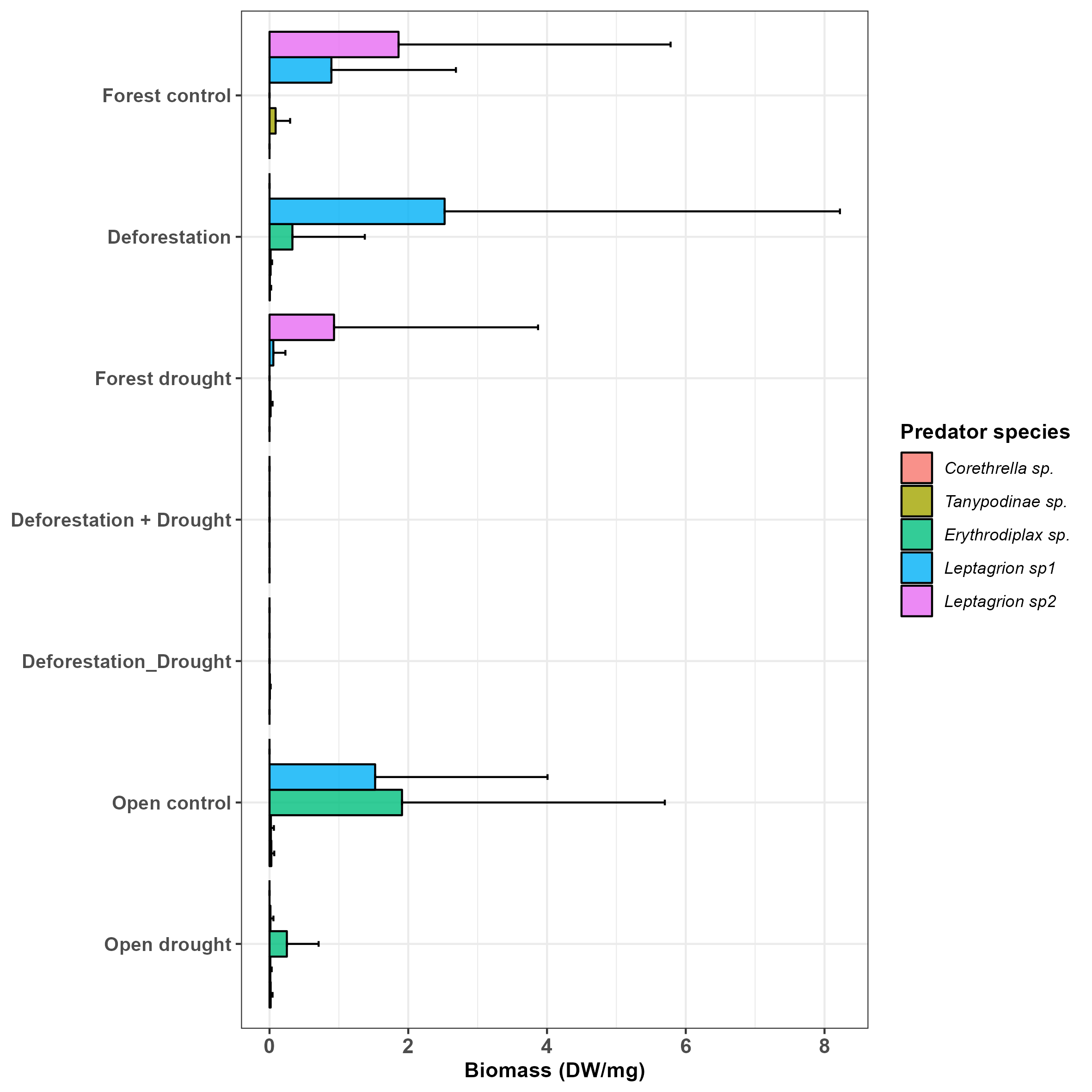

Supplement: Supplemental Information 4 [file peerj-12-17346-s004.png]

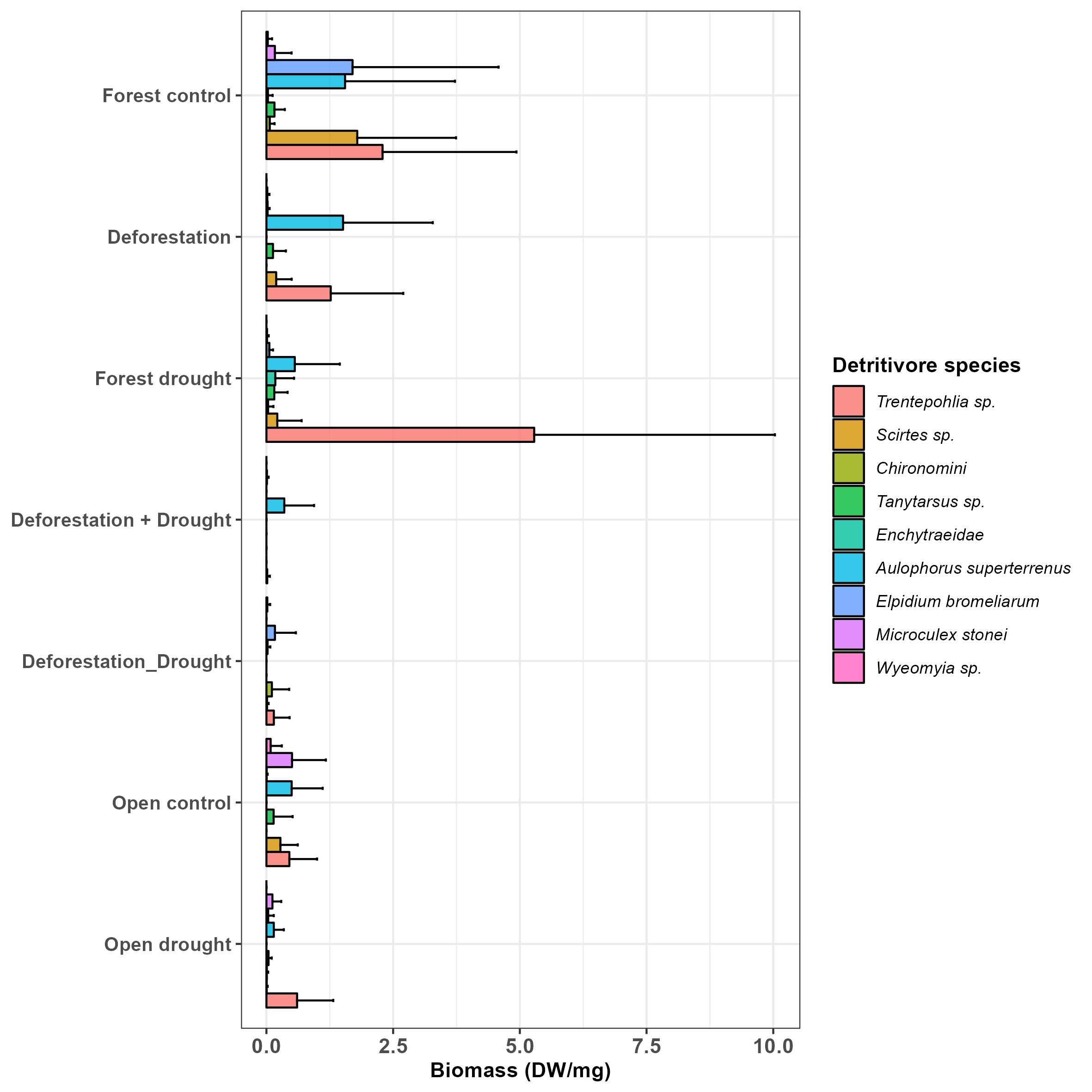

Supplement: Supplemental Information 5 [file peerj-12-17346-s005.png]

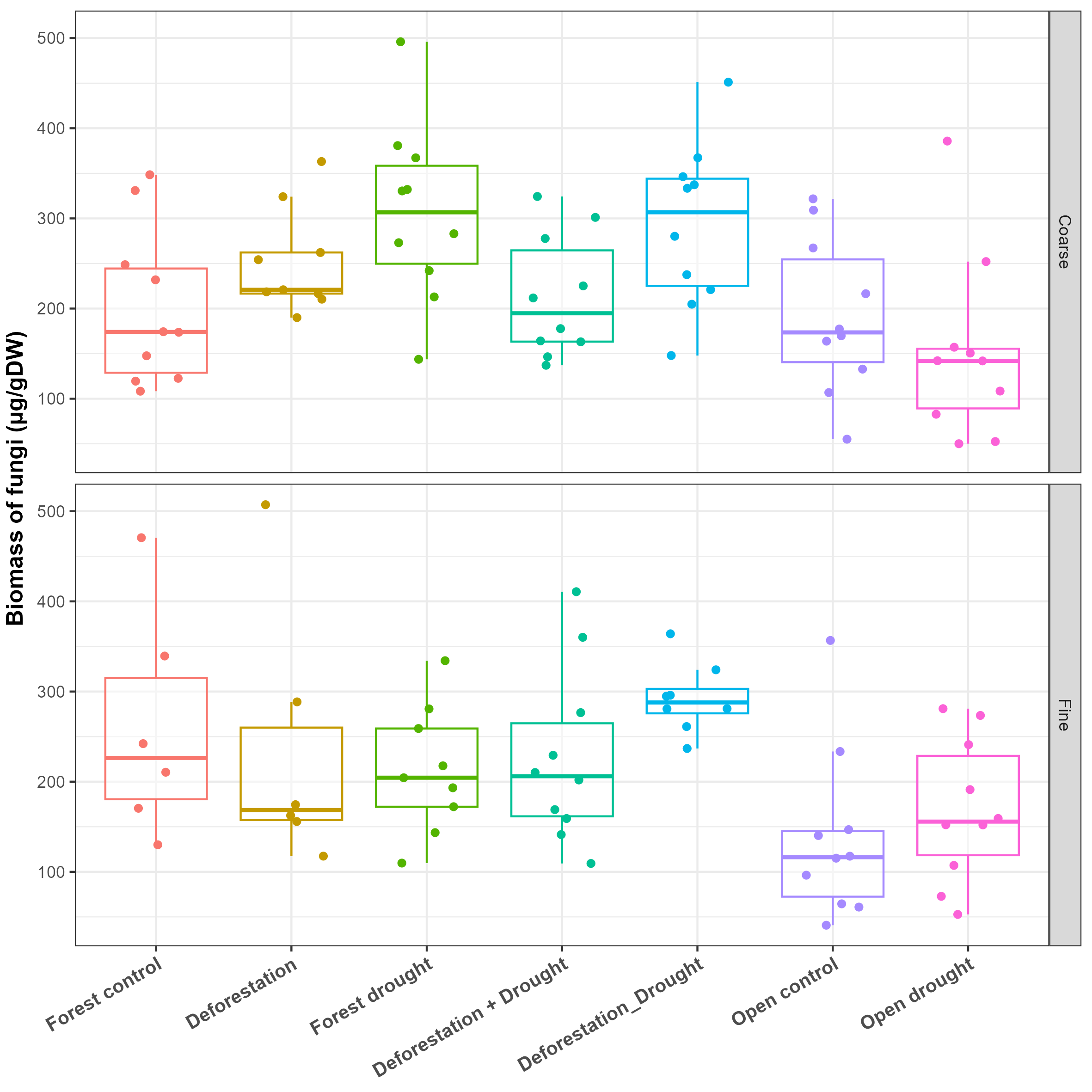

Supplement: Supplemental Information 6 [file peerj-12-17346-s006.png]
